# Applications and limitations of using patient-specific 3D printed molds in autologous breast reconstruction

**DOI:** 10.1007/s00238-018-1430-3

**Published:** 2018-06-16

**Authors:** Stefan Hummelink, Arico C. Verhulst, Thomas J. J. Maal, Dietmar J. O. Ulrich

**Affiliations:** 1 Department of Plastic Surgery, Radboud University Nijmegen Medical Centre, P.O. Box 9101, 6500 HB Nijmegen, The Netherlands; 2 Radboudumc 3D Lab, Radboud University Medical Center, Nijmegen, The Netherlands; 3 Department of Oral- and Maxillofacial Surgery, Nijmegen Medical Center, Radboud University, Nijmegen, The Netherlands

**Keywords:** 3D stereophotogrammetry, Patient-specific template, Breast reconstruction, 3D printed, 3D printing

## Abstract

**Background:**

Over the last years, several techniques have been proposed to improve the outcome of autologous breast reconstruction procedures. One of these innovations describes patient-specific, three-dimensional (3D) printed breast molds for intraoperative use based on 3D stereophotogrammetry. In this article, we want to share our preliminary experiences with producing such templates, its clinical possibilities and limitations in practice.

**Methods:**

Patient-specific templates were designed based on 3D stereophotogrammetry images. The 3D template was fabricated using a 3D printer. During breast reconstruction, the autologous flap was placed inside the printed template to aid the surgeon in determining the shape and volume of the autologous flap creating the desired breast dimensions. Patients were 3D-photographed 6 to 9 months post-operatively.

**Results:**

Three patients with unilateral breast reconstructions showed a width difference of 0.5 cm and mean volume difference of 211 ml between the reconstructed and contralateral breasts. In the three bilateral reconstructed patients, a mean difference in breast width and volume of respectively 0.5 cm and 16 ml was found.

**Conclusions:**

Patient-specific breast templates are inexpensive and relatively easy to design, while being practical and convenient to obtain insight in the dimensions of the desired breast during reconstruction, according to the operating surgeons. Patient selection is however critical, as patients must have sufficient donor volume and/or satisfying breast shape to be able to use the template to its full potential.

Level of evidence: Level IV, therapeutic study.

## Introduction

Over the past decades, the number of breast reconstructions has greatly increased. For reconstructions using autologous tissue, the most commonly applied technique is the deep inferior epigastric artery perforator (DIEP) flap [1]. During this reconstruction, subcutaneous fat and skin from the lower abdomen is transferred as a vascularized free flap to the torso in order to reconstruct the breast [2]. Another method in autologous breast reconstructions is the profunda artery perforator (PAP) flap, where tissue from the inner thigh is transferred to the thorax to be modeled into a breast [3]. Obtaining an esthetically pleasing breast reconstruction correlates with the composed conus and footprint of the reconstructed breast. Finesse and intuition are needed to harvest and establish the ideal flap shape and volume [4]. Providing aid in this process can be beneficial for both surgeon and patient [5].

Pre-operative imaging for breast reconstruction procedures is gold standard in many medical centers. Amongst the imaging modalities is three-dimensional stereophotogrammetry (3D photography). Through this technique, several images of the subject are simultaneously taken using multiple photo cameras under various angles. As the angles between these cameras are known, the position of separate points on the subject’s surface can be calculated, resulting in an accurate 3D surface model of the subject. Apart from elaborately and objectively documenting the patient’s pre-operative status, extra information can be derived from these 3D images. Breast parameters can be objectively quantified and utilized prior to and during the breast reconstruction procedure, improving patient healthcare [6–10].

Amongst the applications utilizing the information obtained through photographing breast is the designing of patient-specific breast molds to facilitate the surgeon in flap shaping [11, 12]. During the breast reconstruction procedure, the autologous tissue can be placed inside the template in such a manner that the flap adapts to the shape. If necessary, adjustments to the free flap can be made based on the template to match the dimensions of the desired breast. Such novel techniques may aid in to manage patient expectations and optimize breast reconstruction outcomes.

In this study, the design and application of templates produced with a 3D printer were investigated for feasibility in a pilot study for both immediate and delayed reconstructions, as well as unilateral and bilateral reconstructions. We share our methods for obtaining such 3D printed mold and our preliminary experiences of the clinical possibilities and limitations in practice, illustrated by three contrasting cases.

## Methods and patients

Six consecutive patients between 31 and 65 years old were included in this feasibility study. Three patients underwent a unilateral, delayed breast reconstruction and three patients received a bilateral reconstruction (one immediate, one delayed reconstruction, and one delayed with the use of tissue expanders). All patients followed a standard pre-operative imaging protocol which consisted of a computed tomography angiography (CTA) scan for evaluation and 3D planning of perforators, and 3D photography of the torso for clinical documentation [13, 14]. 3D images of the torso were obtained using a commercially available multi-camera set-up, 3dMD Body (3dMD, Atlanta, USA). The system consists of four pods with a total of 12 cameras aimed at the patient’s torso at various angles. During image acquisition, the patient was standing in front of the cameras with the hands positioned at the hips for reproducible results [15]. The associated software with the 3D stereophotogrammetry system automatically created a 3D surface model from the captured images. The computer model obtained through 3D photography was exported and loaded into 3D software Autodesk 3ds Max (Autodesk Inc., USA) to start the process of creating a 3D template to be used during breast reconstruction surgery.

In the case of a unilateral breast reconstruction, the contralateral breast was delineated and virtually isolated from the torso. After mirroring along the vertical axis, it was transposed to the affected site in the 3D software. Alterations to the edges of the virtually isolated breast were made to ensure the contours would follow the thorax wall. Theoretically, any modification to the virtual breast could be made at this point, such as a virtual ptosis reduction, which would be reflected in the 3D printed breast mold.

In the case of an immediate bilateral breast reconstruction, the most esthetically pleasing breast was selected to be mirrored to the contralateral side for symmetry purposes following the same principles as the unilateral breast reconstruction 3D mold creation process. Theoretically, both breasts could be virtually isolated and produced into a breast template, if patient wishes to approach her natural asymmetry in the pre-mastectomy situation.

In the case of a delayed bilateral breast reconstruction, the absence of a pre-operative 3D photographic model was encountered. No 3D pre-operative information was available on breast volume or shape, as mastectomy had already been performed. In consultation with the plastic surgeon performing the breast reconstruction, a virtual breast was crafted suiting the patient’s posture.

**Fig. 1:**
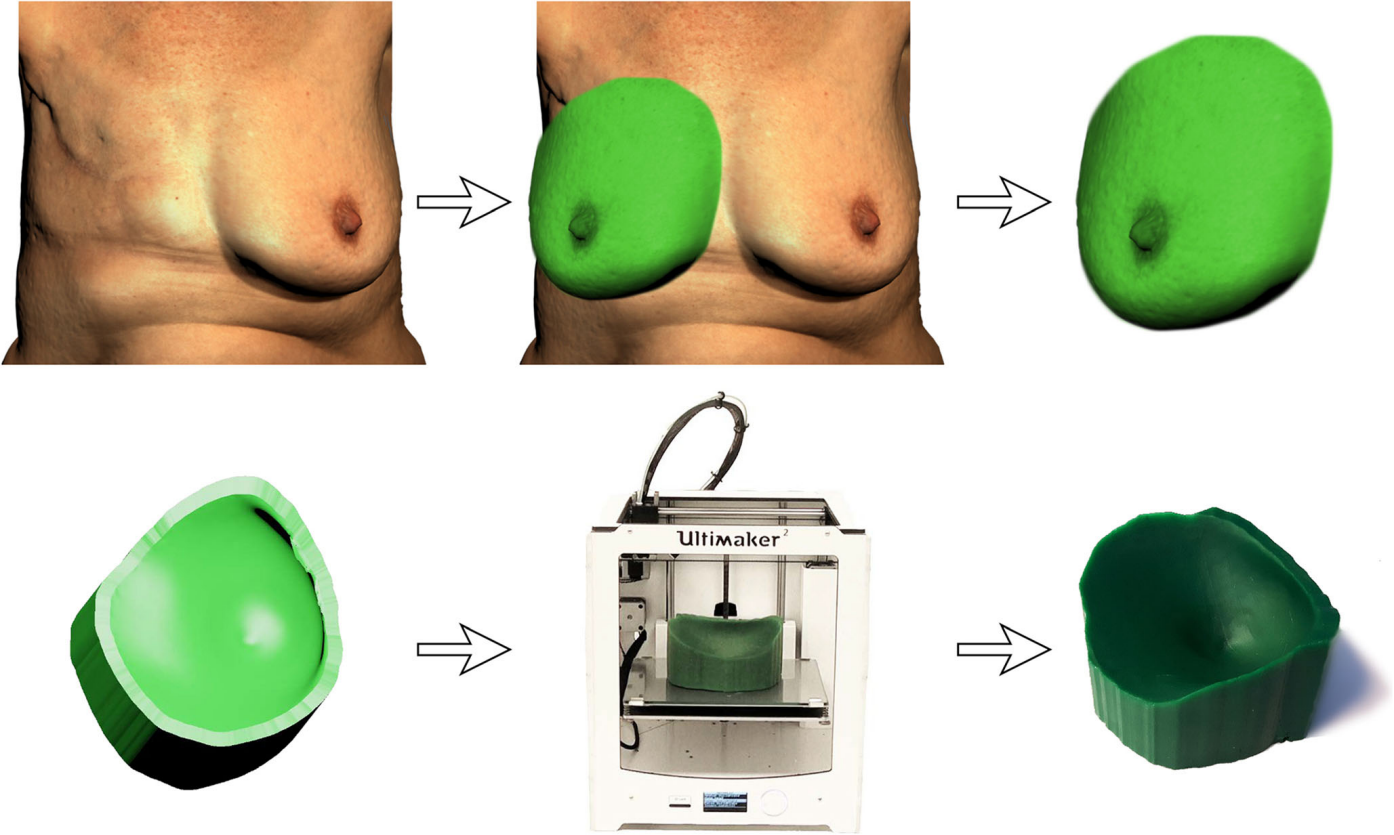
Design of a patient-specific template for a unilateral breast reconstruction. The contralateral breast is virtually isolated and mirrored based on 3D photography. Extrusion of the outer rim of the virtual breast provides a flat base. Finally, the design is printed using a 3D printer

The finalizing steps of creating a breast mold for either breast reconstruction setting incorporated the ventral extrusion of the outer rim of the breast contour. This provides the breast mold to be placed on a flat surface in such a manner that the flap can be conveniently positioned inside the template. The template of the wall was thickened to 4 mm to providing sufficient strength during intraoperative use. The final step in virtually modeling the surgical breast template was to clean up and smoothen the 3D design. A general overview of the 3D printing design procedure is shown in Fig. 1.

The model was imported into 3D printing software Cura (Ultimaker B.V., the Netherlands), where support structures were added to prevent the model from collapsing during the printing process. The mold was printed with an Ultimaker 2 (Ultimaker B.V., the Netherlands) using Polylactic Acid (PLA) filament on default printer settings. After 3D printing of the template, the supporting material was removed manually.

Once in an operative setting, the 3D printed mold was placed in a sterile plastic sleeve to be used for the fitting of the free flap. Prior to anastomosis, the flap was positioned in this sterile covered template, where the contours of the free flap could be traced with a marker pen along the 3D printed mold, and sutures can be placed to maintain flap shape.

As part of standard photo documentation, 3D images were captured of all patients 6 to 9 months post-operatively. Final breast volume and width on these images was assessed using Autodesk 3ds Max software.

To gain more insight into the possibilities and limitations of patient-specific 3D printed breast templates, three contrasting cases will be discussed further.

### Patient cases

Case 1, aged 37, presented herself with a palpable malignant tumor in the right breast. She was diagnosed with BRCA gene mutation for which a bilateral, preventive ablation with direct subpectoral tissue expander placement was performed. In a later stage, the breast reconstruction would be completed via a DIEP flap. After completing the periodic filling of the tissue expanders, the final breast volume measured 490 cm^3^ each. As her breasts were asymmetrically positioned due to the prior surgery, the right breast formed the basis for the 3D printed templates.

Case 2 was predisposed with a BRCA-1 gene mutation for which a bilateral preventive ablation was planned along with a direct reconstruction. The PAP flap technique was selected for this 30-year-old woman, as insufficient abdominal tissue was available. Although the patient was satisfied with her breast volume, she would like the ptosis of her breast to be altered.

Case 3 had undergone a unilateral breast-saving lumpectomy as a result of a tumor in the right breast when she was 49 years old. She presented herself with a retracted scar, displaced nipple, poor skin quality and lymphedema of her right arm after radiotherapy and chemotherapy. It was determined that she was eligible for a unilateral DIEP flap, alongside with a vascularized autologous lymph node transfer to be placed within the axilla.

## Results

In six patients, pre-operative 3D photographs were taken on which a patient-specific 3D printed mold was designed to be used during the breast reconstruction. Six to 9 months post-operatively 3D photographs were repeated in order to calculate the breast width and volume between of reconstructed breast. Patients who had unilateral reconstructions, a width difference of 0.5 cm and mean volume difference of 211 ml between the reconstructed breast and contralateral side were found. For bilateral reconstructed patients, a mean difference in breast width and volume of respectively 0.5 cm and 16 ml was found. Results of these postoperative measurements are shown in Table 1.

**Table 1:** Breast volumes and breast widths derived from 3D stereophotogrammetry 6 to 9 months after surgery with utilization of the 3D printed template

Case	Reconstruction	Volume (cc)			Width (cm)		
		Left	Right	Difference	Left	Right	Difference
1	Bilateral	619	603	16	20	19	1
2	Bilateral	279	263	16	16	16	0
3	Right	580	864	284	18	18.5	0.5
4	Bilateral	420	403	17	19	19	0
5	Right	803	620	183	19.5	19.5	0
6	Left	462	629	167	17	18	1

### Patient cases

#### Case 1

**Fig. 2:**
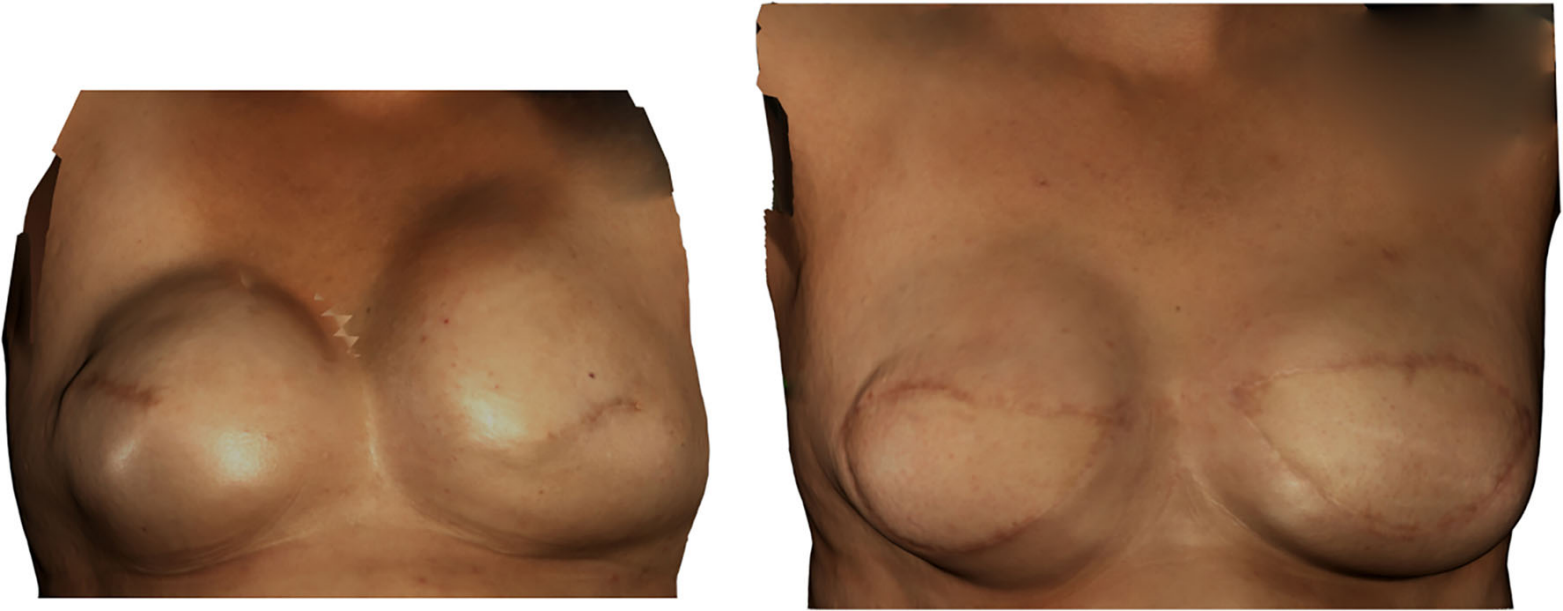
Case 1. Status after bilateral preventive ablation with completed tissue expanders filling. Left: tissue expander in situ prior to DIEP flap reconstruction. Right: 9 months after DIEP flap reconstruction, prior to secondary corrections

After completing filling procedures, her tissue expanders contained 490 cm^3^ saline fluid. The expanders were removed and the expanded skin pocket was padded with the (partially) de-epithelialized DIEP flap. Clinical photos can be seen in Fig. 2. Nipple reconstruction and secondary corrections were performed at a later stage.

#### Case 2

**Fig. 3:**
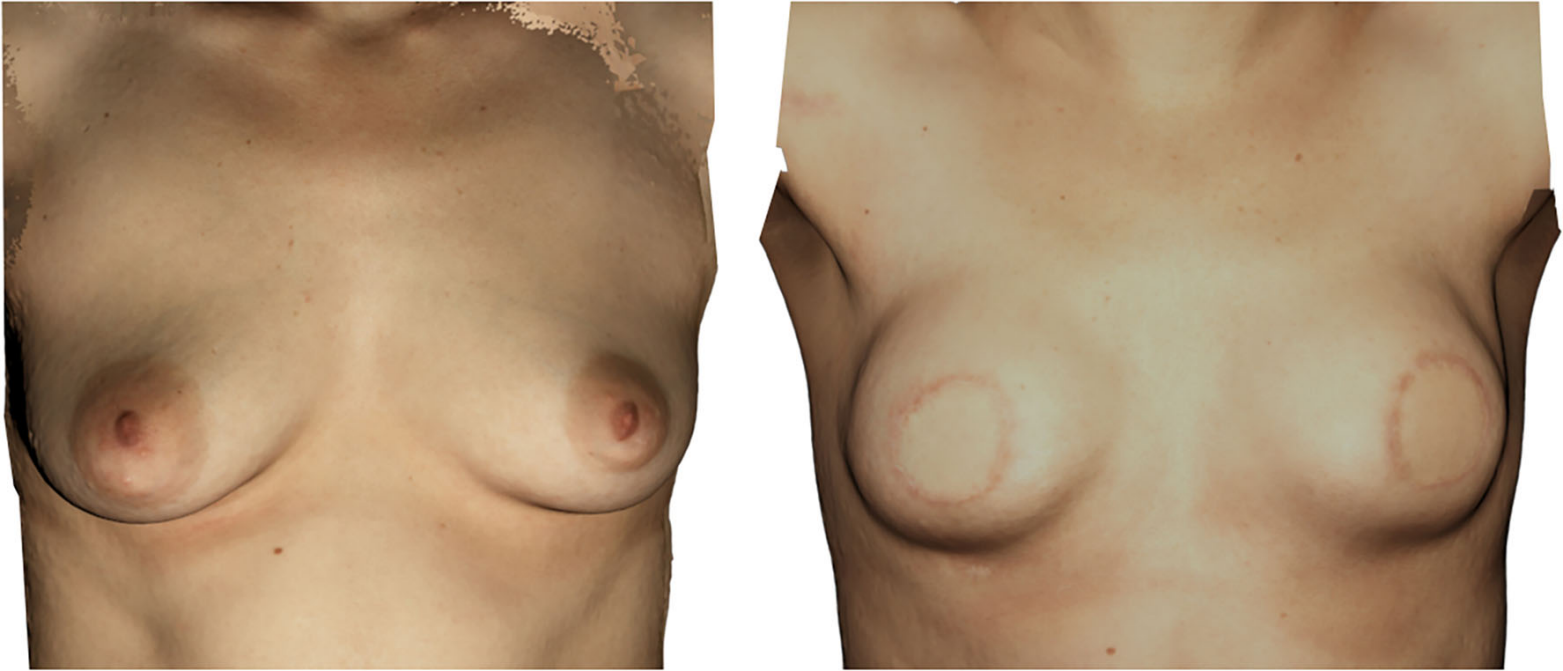
Case 2. Bilateral preventive ablation with a direct breast reconstruction using the PAP flap technique. Left: pre-operative situation with mild volume defect in the upper pole. Right: 6 months after PAP flap reconstruction, prior to secondary corrections

The aim of this 3D printed mold was to recreate the patient’s breast shape prior to ablation. In consultation with the operating surgeon no modification was made to the 3D printed mold, despite the mild upper pole volume defect. As per patient request, the required upper pole volume was increased intraoperatively by surgeon experience. The pre-operative situation and 6-month post-operative breast reconstruction results are shown in Fig. 3 prior to nipple reconstructions.

#### Case 3

**Fig. 4:**
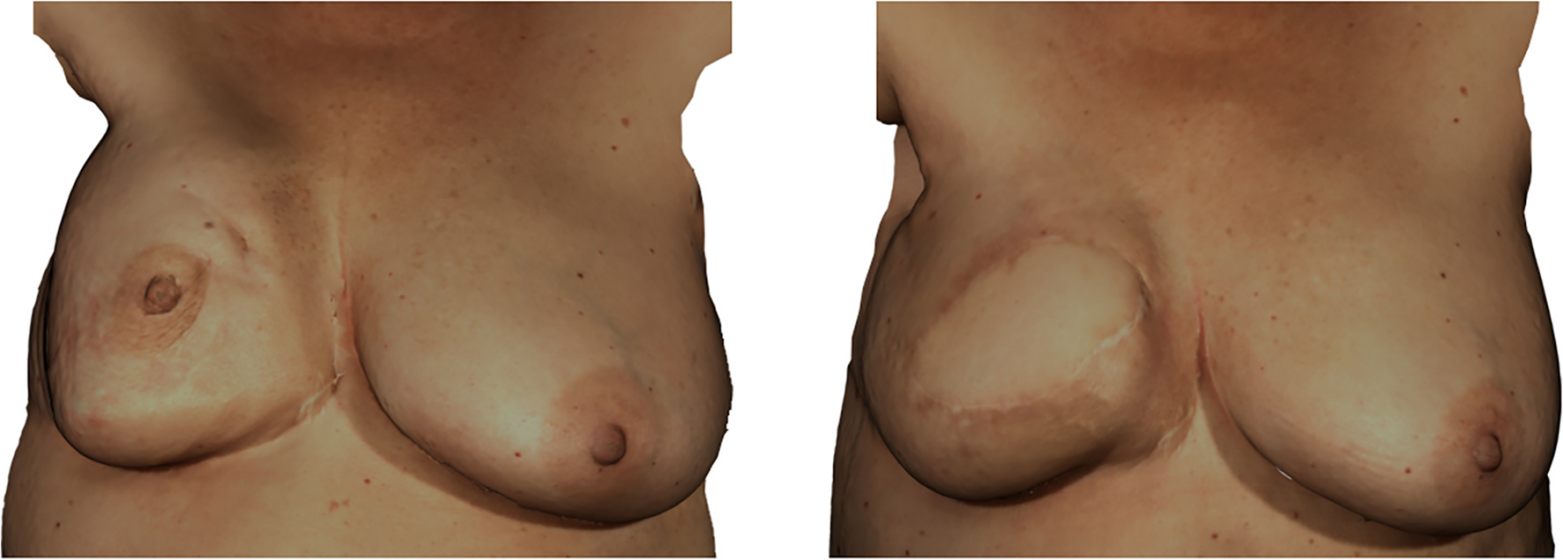
Case 3. Unilateral DIEP flap reconstruction with insufficient donor site volume. Left: pre-operative situation after lumpectomy, radiotherapy and poor skin quality. Right: 6 months after DIEP flap reconstruction, prior to secondary corrections

A 3D printed template was produced for the harvested flap, based on mirroring of the contralateral side. However, total flap volume to be obtained from the donor site proved to be insufficient. In a secondary procedure, breast volume was increased through lipofilling; additionally, the contralateral breast was reduced and lifted. Due to insufficient donor site volume, the 3D printed mold was of little added value for volume assessment in this case and was used for determining the width and placement of the breast reconstruction. Photos of the reconstruction result prior to the second symmetrising procedure can be found in Fig. 4.

## Discussion

Our preliminary clinical experience with the usage of patient-specific 3D printed breast molds has been described in this paper and illustrated by three diverse clinical cases. Using already available techniques and equipment, 3D printed breast templates were created for patients undergoing both unilateral and bilateral breast reconstructions.

In this feasibility study, the post-operatively recorded breast volumes and width varied between patients. In the three bilateral breast reconstructions, minimal post-operative breast volume difference between the reconstructed breasts was measured. The three unilateral reconstructions showed however a substantial deviation in breast volume between both breasts, as either the patient had insufficient donor site volume, or her wishes were to get a ptosis reduction in the contralateral side. In all unilateral patients, secondary corrections of the contralateral breast were performed to get more symmetric results.

For the template to be utilized to its full potential, patient selection for the creation of 3D printed breast molds is crucial. Patients should be satisfied with their natural breasts, or unaffected side. An adequate amount of adipose tissue should be obtainable from the donor site. By pre-operatively comparing fat volume measurements on the pre-operatively performed CTA scan with breast volume measurements derived from the 3D photo, one can acquire insight in whether the desired volume can be reached within one procedure. Additionally, in case of a greater surplus of the donor site compared to the breast volume, a cover on top of the 3D printed breast template could be beneficial to encapsulate the total breast shape.

Unilateral breast reconstruction patients may have a tissue expander in situ. When designing 3D printed breast mold, it should be noticed that the expanded breast pocket may not correlate with the shape of the natural breast. Measuring the total breast volume and subtracting the volume injected into the tissue expander can provide information regarding the thickness of the mastectomy skin flap. When mirroring the contralateral side design steps can be taken into consideration to correct for locally present volume from the expanded mastectomy flap. To manage patient expectation, one may even speculate on the idea that in consultation with the patient and plastic surgeon the breast reconstruction is virtually shaped, of which a mold is printed.

In both unilateral and bilateral delayed breast reconstructions, multiple factors are at play when determining the final shape of a reconstructed breast. The thickness of the mastectomy flap and quality of the skin pocket are of essence in the decision regarding how the reconstruction should be performed. The skin paddle from the autologous flap is de-epithelialized and trimmed to replace the native skin of insufficiently quality by donor skin from the autologous flap. How much skin paddle should be utilized in autologous flap reconstruction should be determined prior to placement of the flap in the breast mold, as the overall contour and shape of the reconstructed breast are influenced by these decisions.

The technique of 3D stereophotogrammetry is part of standard clinical workup for all our breast reconstruction patients. Also, an Ultimaker 2 was already available in our hospital for research purposes. The template was designed within 15 min and printing time was about 16 h. Using a sterile sleeve as protective packaging instead of a sterile printed mold is more economical, without being impractical during surgery. The Autodesk 3ds Max software used for creation was available as already available license to our department; however, free alternative software such as MeshMixer (Autodesk Inc., USA) can also be used for the purpose of designing 3D models. Therefore, this research was conducted without purchasing additional hardware. The total added cost for this innovation in autologous breast reconstruction is estimated at 20 euro, a negligible addition in comparison to the total breast reconstruction procedure costs.

According to surgeon’s opinion, 3D printed breast templates could be a useful tool during the operation for assessing the flap volume, shape, and orientation. They expressed it was convenient in practice and aided in visualizing the final shape of the reconstructed breast. Especially less experienced surgeons may benefit from the usage of the proposed 3D printed template. The most potential of this technique would lay in unilateral patients who are satisfied with the shape of their unaffected breast and have adequate donor site volume. In bilateral reconstructions a symmetric breast reconstruction may be obtained more conveniently. These hypotheses would need to be tested in a larger study. Future studies will focus on clinical outcomes such as patient satisfaction, surgery time, breast volume, size and symmetry between breasts within a larger, patient-selected population. As design and printing can be done relatively quickly at low costs, only a minor improvement in outcome would already result in a cost-effective clinical tool.

## Conclusions

Recreating the female breast in an autologous reconstruction requires insight and skill. Through 3D stereophotogrammetry and 3D printing, a patient-specific template can be created, supporting the surgeon in the intraoperative decision-making of breast shaping. Creating the breast template can be done at low costs and relatively fast. For both unilateral and bilateral breast reconstructions, in a direct or delayed setting, a breast mold could be considered as a useful and cheap addition to the autologous breast reconstruction procedure. However, if the patient has insufficient donor site volume available, or is unsatisfied with the appearance of her natural breast, the process of fabricating a 3D printed template is of little added value. Therefore, patients should be pre-selected for utilizing this technique.
